# The association between the dynamics of COVID-19, related measures, and daytime population in Tokyo

**DOI:** 10.1038/s41598-022-06716-4

**Published:** 2022-02-23

**Authors:** Takenori Yamauchi, Shouhei Takeuchi, Mitsuo Uchida, Masaya Saito, Akatsuki Kokaze

**Affiliations:** 1 Department of Hygiene, Public Health and Preventive Medicine, School of Medicine, Showa University, Tokyo, Japan; 2 Department of Nutrition Science, Faculty of Nursing and Nutrition, University of Nagasaki, Nagasaki, Japan; 3 Department of Public Health, Graduate School of Medicine, Gunma University, Gunma, Japan; 4 Department of Information Security, Faculty of Information Systems, University of Nagasaki, Nagasaki, Japan

**Keywords:** Epidemiology, Viral infection

## Abstract

In Japan, a novel coronavirus has been prevalent since January 2020. The Japanese and local governments have implemented various measures, including declaring a state of emergency, according to the epidemic situation in each region. This study estimated the effective reproduction number (*R*_t_) using the number of confirmed positive cases and positivity rates in Tokyo and examined the association between *R*_t_ and the rate of increase/decrease in the number of people across 12 sites. In Tokyo, there were five waves in which *R*_t_ was persistently estimated as approximately 1.0. The fourth and fifth waves started under the declaration of the state of emergency and coincided with an increase in the number of people. However, the contribution of the number of people to *R*_t_ was inconsistent, even when the number of people was of the same magnitude. A possible reason for this is difference in the countermeasures content, as the impact of vaccination was considered to be minor at the time. Where vaccination is insufficient, the wave is terminated by controlling the number of people leaving their homes. It is suggested that infection could be controlled more efficiently, depending on the content of the countermeasures.

## Introduction

Coronavirus disease (COVID-19) is an infection caused by the novel coronavirus severe acute respiratory syndrome coronavirus 2 (SARS-CoV-2) that has spread worldwide since the end of 2019. Confirmed cases and deaths were reported to be over 225 million and over 4.6 million, respectively, on 16 September 2021^1^. In Japan, the index case was reported as a Chinese national living in Kanagawa Prefecture on 15 January 2020^2^. To take measures within the framework of the Infectious Diseases Control Law to prevent the spread of infection, COVID-19 was designated as an infectious disease on 28 January 2020, and requests for measures such as restricting access to buildings and refraining from going outside were implemented^3^. In addition, COVID-19 was designated as “a new influenza and other infectious disease” from 3 February 2021.

On 16 September 2021, the numbers of PCR-positive cases and deaths in Japan were 1,652,919 and 16,952, respectively^4^. From the end of 2019, various measures were implemented to prevent the spread of infection. For example, elementary, junior high, high, and special needs schools were temporarily closed^5^. The Japanese government declared the first state of emergency (SoE) on 7 April 2020^6^. In Tokyo, the Japanese government declared an SoE a total of four times^7^. Refraining from going out unnecessarily, shortening the business hours of stores, and restricting the holding of events were included in the emergency measures implemented. From 21 June 2021 to 11 July 2021, the Tokyo Metropolitan Government (TMG) implemented priority measures to prevent the spread of coronavirus infection, which required the same measures as those under the emergency declaration. In addition, the TMG requested restaurants and karaoke parlours serving alcoholic beverages to shorten their business hours from 3 August 2020 to 15 September 2020^8^. Similar measures were taken from 28 November 2020 to 7 January 2021. Furthermore, to infection control measures, the government also launched the “Go To” campaign, an economic policy aimed at revitalising an economy that had been exhausted by the pandemic restrictions. The effective reproduction number (*R*_t_) is defined as the average number of secondary infections produced by a typical infected person in a particular population^9^. The number of infected persons was expected to increase when the *R*_t_ was greater than 1.0 and decrease when it was less than 1.0. Therefore, *R*_t_ is considered useful for assessing the transmissibility of infectious diseases^10^. In this study, we estimated the *R*_t_ in two ways, and examined the association between the *R*_t_ and the timing of countermeasures and events. Then, we aimed to clarify the contribution of human flow at 12 points in Tokyo to the *R*_t_.

## Results

### Infection status in Tokyo

**Figure 1:**
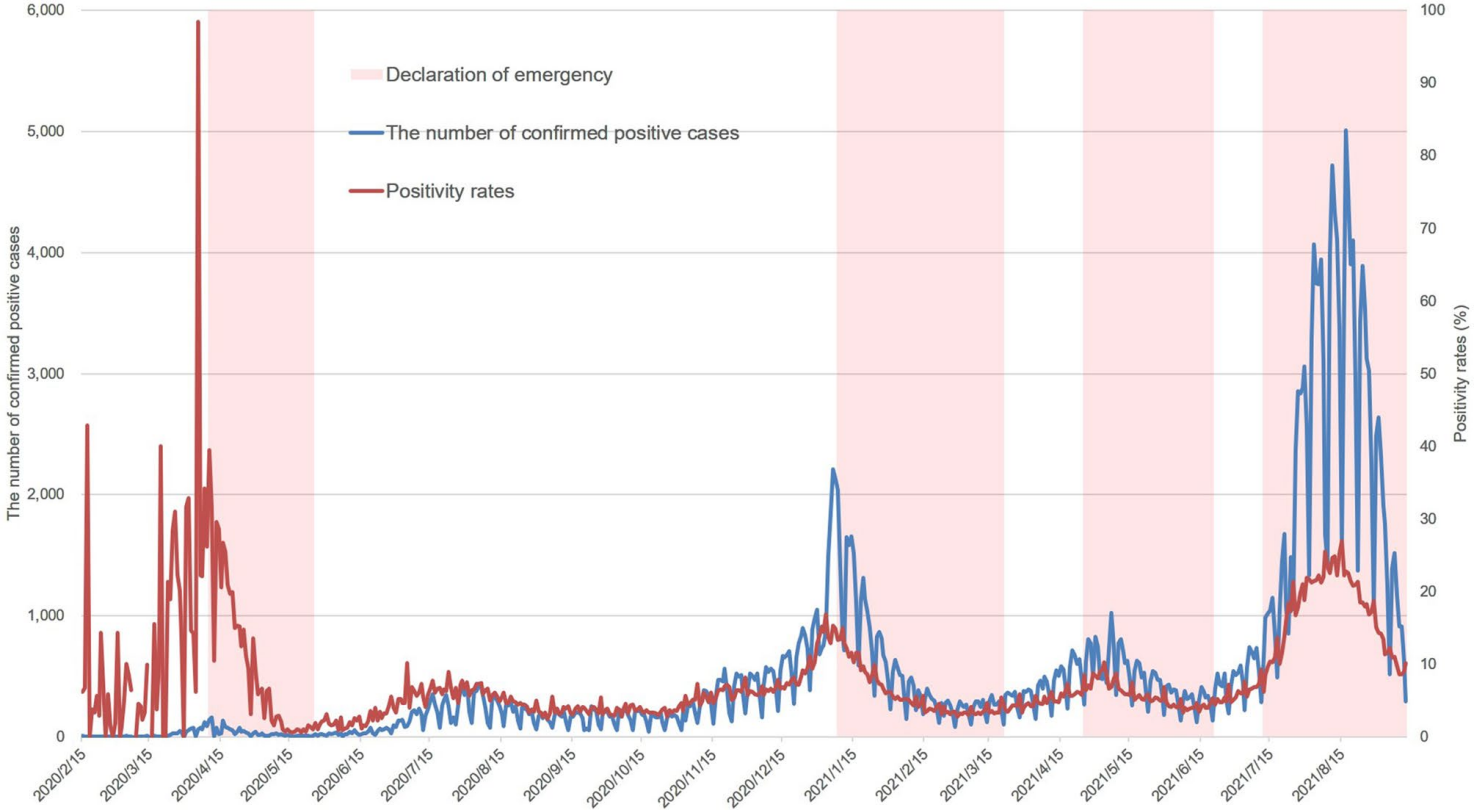
Time series transition of the number of confirmed positive cases and positivity rates and the period of emergency declaration in Tokyo. The daily number of confirmed positive cases (blue line) and positivity rates (red line) are shown. The periods of the emergency declaration are shown in pink.

The number of confirmed positive cases (cPCs) and the positivity rates (PRs) in Tokyo are shown (Fig. 1). According to the number of cPCs, five waves of coronavirus infection were observed between February 2020 and September 2021. These waves peaked around mid-April 2020, from the end of July to the beginning of August 2020, at the beginning of January 2021, in mid-May 2021, and mid-August 2021. Weekly periodical increases and decreases were observed in the number of cPCs with respect to the number of PCR tests conducted (inspections), which decreased considerably over weekends and holidays. A similar trend of periodical fluctuation in relation to inspections was observed for the PR, although it was not as notable as for the number of cPCs. With the exception of the first wave, in which the number of cPCs was low, the pattern observed in the time series of cPCs and PRs were generally similar. When focusing on the relationship between the declaration of an SoE and the trend in the time series data, both the number of cPCs and PRs consistently decreased during the declaration period, except during the fourth wave. To estimate the distribution of the period from onset to being confirmed positive, the 95% data range was used, since outliers were identified in the early stage of the epidemic. Among the fitted distributions, the distribution with the highest likelihood was the Weibull distribution with a mean and standard deviation of 2.8 and 2.0, respectively, and it was used for back projection.

### Ratio and trend of the increase/decrease in the number of people at 12 sites

**Figure 2:**
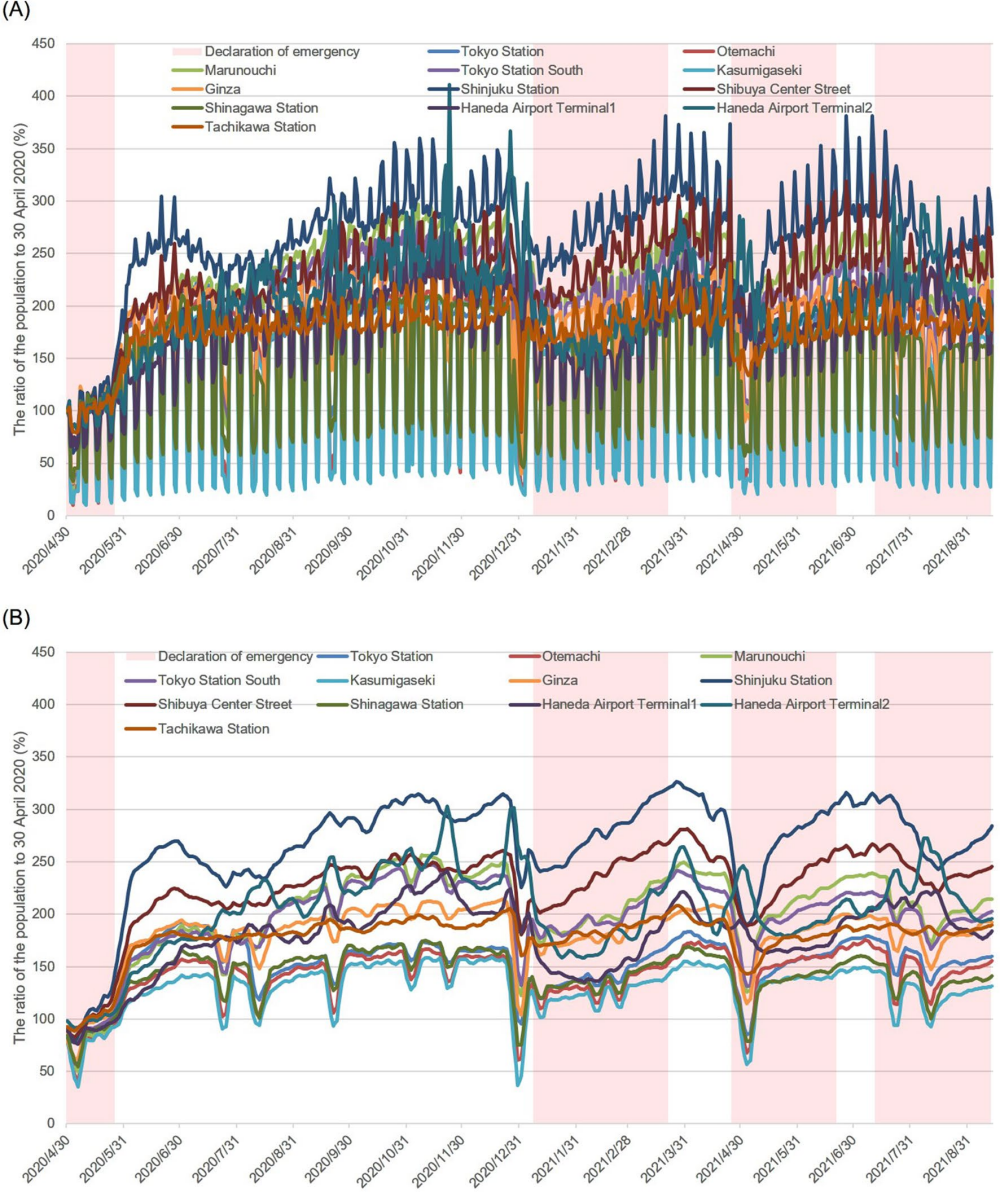
The ratio of the population at 12 sites and the period of emergency declaration. The ratio of the population to 30 April 2020 at 12 sites (i.e. Tokyo station, Otemachi, Marunouchi, Tokyo station south, Kasumigaseki, Ginza, Shinjuku Station, Shibuya Center Street, Shinagawa Station, Haneda Airport Terminal 1, Haneda Airport Terminal 2, and Tachikawa station) is shown. Raw data are shown in (**A**), and only the trend components are shown in (**B**). The periods of the emergency declaration are shown in pink.

The ratio of the increase/decrease in the number of people at the 12 sites is shown in Fig. 2A. Figure 2B shows the trend component only, excluding the periodic and residual components. In the second and third emergency declarations, there was a significant decrease in the number of people just before and after the declaration, and then a gradual increase in the number of people was observed at all locations. In comparison, in the fourth SoE, the number of people infected decreased about a week after the declaration. Immediately after the decrease, an increase was observed before it decreased again. Haneda Airport (HA) Terminal 2 showed an increasing trend in the number of people until 7 August after which it gradually decreased. No sharp increase was observed, in comparison to the period after the first declaration of the SoE was lifted, when there was a sharp increase. The increase in the number of visitors at HA Terminal 2 generally coincided with consecutive holidays. Since the time-series trends at some of the 12 sites were similar, we grouped these by factor analysis. The results are shown in Table 1. Because two eigenvalues exceeded 1.0, two factors were selected. Kasumigaseki, Otemachi, Shinagawa Station, and Tokyo Station were considered as one group, in which loadings of Factor 1 were large and loadings of Factor 2 were quite small. Similarly, Ginza, Marunouchi, and Tokyo Station South, which have large loadings of Factor 1 and moderate loadings of Factor 2, were considered as one group. We also considered Shibuya Center Street, Tachikawa Station, and Shinjuku, which had large loadings of Factor 2 and small loadings of Factor 1, as one group. HA Terminals 1 and 2 were not considered groups. Therefore, we were able to classify the data from 12 locations into five groups in total. For the sake of sensitivity analysis, we used three factors, but the results of the grouping did not change (data not shown).

**Table 1:** Factor analysis of the population ratio of Tokyo.

Area	Factor 1	Factor 2
Kasumigaseki	1.03	−0.23
Otemachi	1.03	−0.20
Shinagawa Station	1.01	−0.12
Tokyo Station	0.98	−0.05
Ginza	0.93	0.18
Marunouchi	0.91	0.22
Tokyo Station South	0.86	0.31
Shibuya Center Street	−0.06	1.00
Shinjuku Station	0.03	0.98
Tachikawa Station	−0.12	0.99
Haneda Airport Terminal 1	0.59	0.36
Haneda Airport Terminal 2	0.14	0.57

### Daily *R*_*t*_, its 95% credible interval, and epidemic acceleration period (EAP) and waves

**Figure 3:**
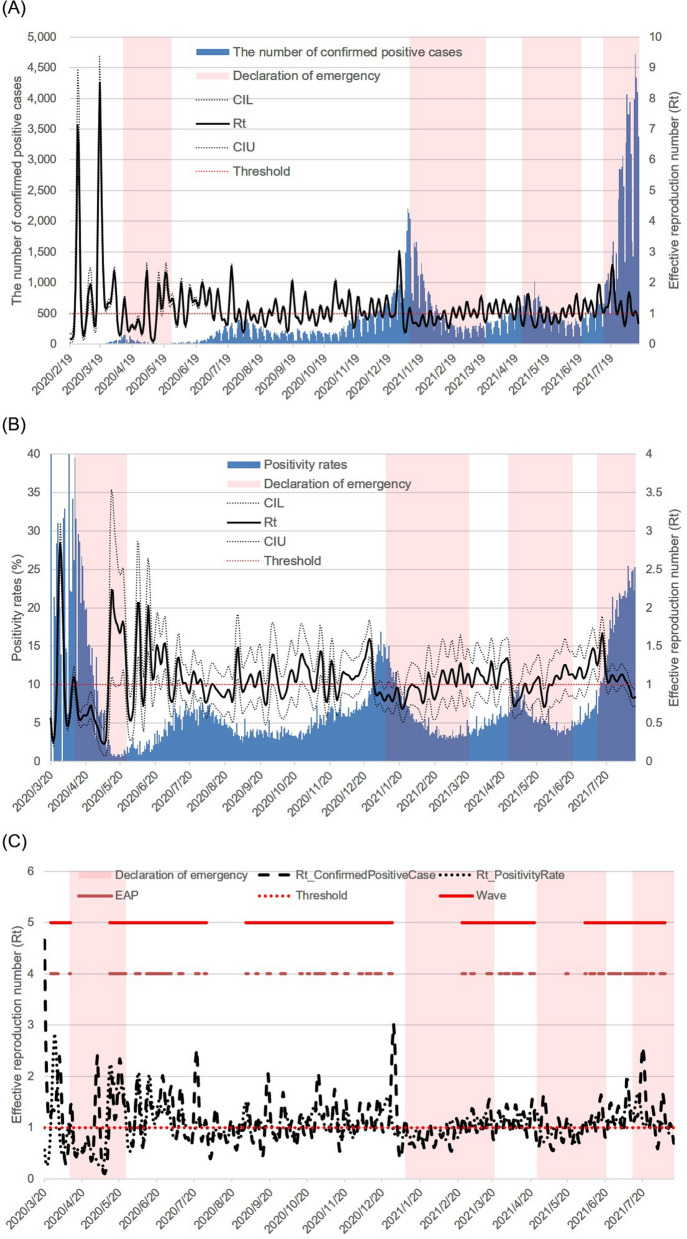
Two effective reproduction numbers, Epidemic Acceleration Period and waves. The estimated effective reproduction number (*R*_t_) and epidemic curve are shown in (**A**, **B**). In (**A**), the number of confirmed positive cases (blue bar), the *R*_t_ (black line), the lower 95% credible interval limit of the *R*_t_ (95% CIL) (black dotted line), the upper 95% credible interval limit of the *R*_t_ (95% CIU) (black dotted line), the threshold for both the *R*_t_ (1.0) (red dotted line), and the periods of emergency declaration (pink dotted line) are shown. The only difference between (**A** and **B**) is that the blue bar indicates the positivity rate. In (**C**), the *R*_t_ estimated by data on the number of confirmed positive cases (black dashed line), the *R*_t_ estimated by data on positivity rates (black dotted line), epidemic acceleration period (EAP) (orange line), wave (red line), the threshold for both *R*_t_ (1.0) (red dotted line), and the periods of emergency declaration (pink dotted line) are shown.

**Figure 4:**
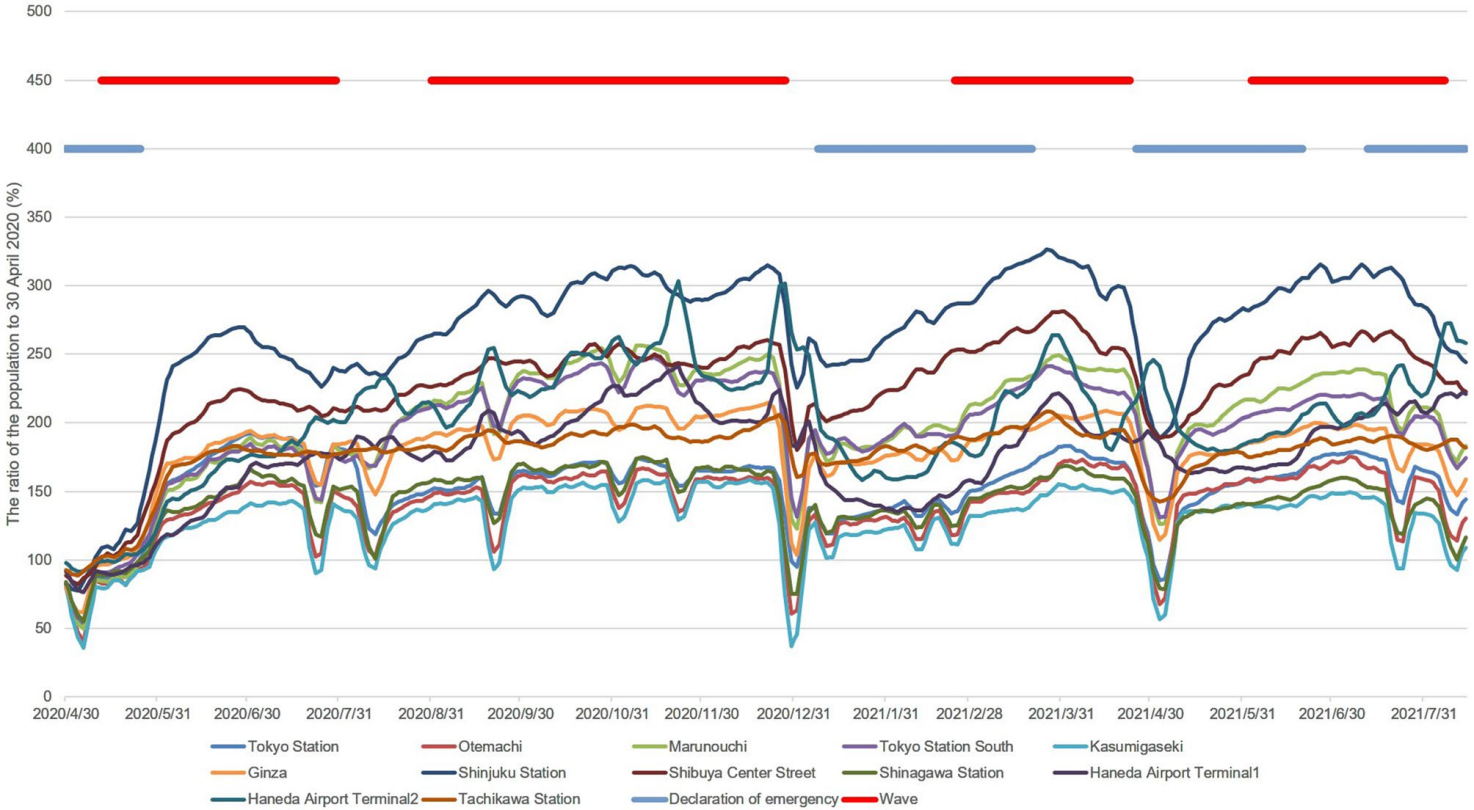
Trends in the number of people at 12 sites, the period of emergency declarations and the period of waves. Only the trend component of the number of people at 12 sites (thin lines) and the period of emergency of declaration (thick blue line) and wave (thick red line) are shown.

The *R*_t_ and 95% credible intervals estimated from two data points, the number of cPCs and PRs, are shown in Fig. 3A and B. The EAP and waves are shown in Fig. 3C. From the distribution of the EAP, the periods of the first, second, third, fourth, and fifth waves in Tokyo were defined as 25 March to 10 April 2020, 12 May to 30 July 2020, 31 August to 28 December 2020, 23 February to 23 April 2021, and 3 June to 7 August 2021, respectively. The trend components of the increase/decrease in people at 12 locations, the period of emergency declarations, and the period of the waves are shown in Fig. 4. It seemed that a new wave had started before the first, second, and third emergency declarations were lifted. The data reflect the start of a new wave when the number of people increased above a certain level. This is especially noticeable in the second and third emergency declarations.

### Contribution of the number of people to *R*_*t*_

**Figure 5:**
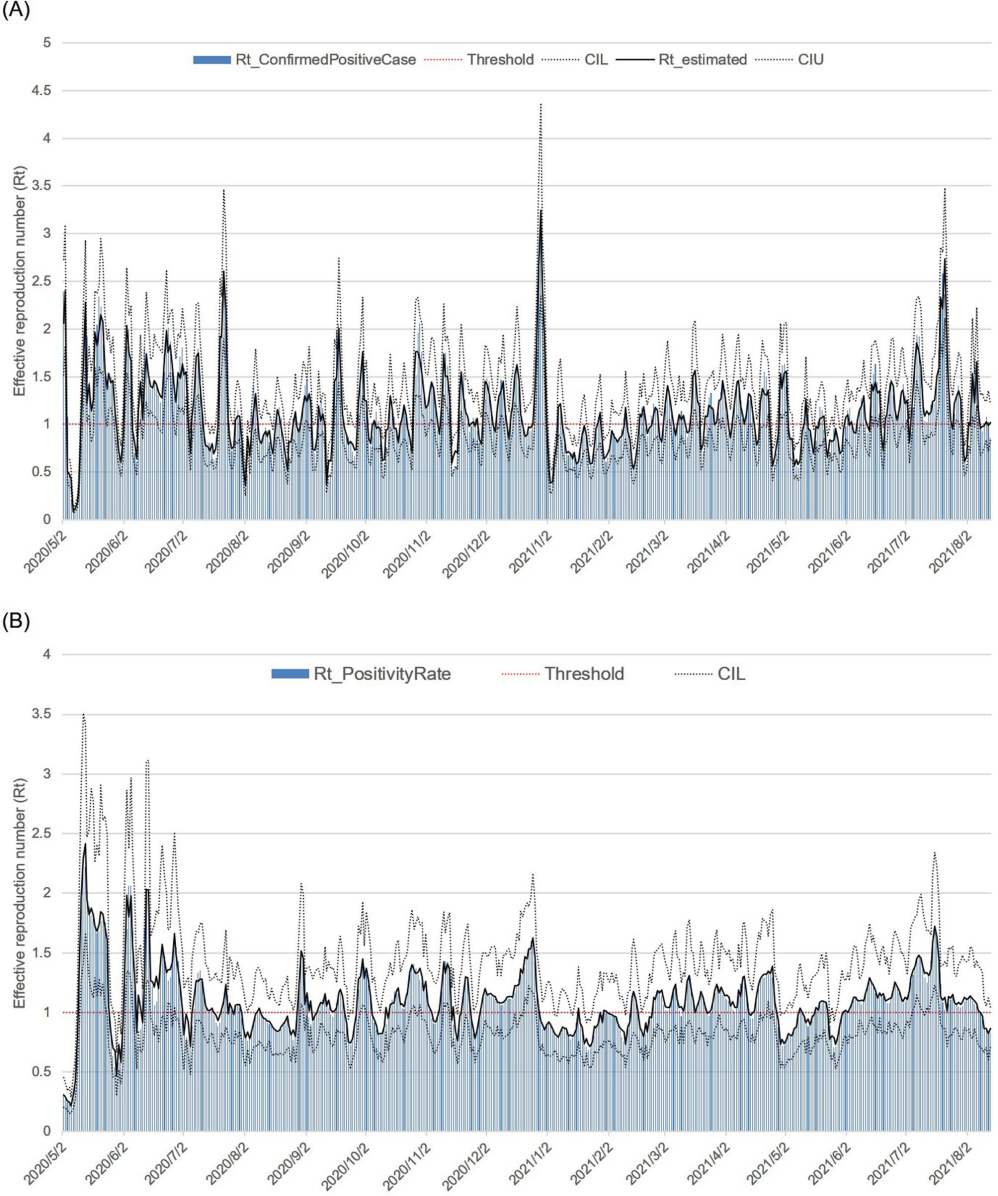
Estimates obtained by applying Eq. (2) to the number of people and effective reproduction number. The fit between the effective reproduction number (*R*_t_) and the estimate obtained by Eq. (2) is shown. In (**A**), all values were estimated using the number of confirmed positive cases. The *R*_t_ estimated by Eq. (1) (blue bar), the threshold for both the *R*_t_ (1.0) (red dotted line), the *R*_t_ estimated by Eq. (2) (black line), the lower 95% credible interval limit of the *R*_t_ estimated by Eq. (2) (95% CIL) (black dotted line), and the upper 95% credible interval limit of the *R*_t_ estimated by Eq. (2) (95% CIU) (black dotted line) are shown. The only difference between (**A, B**) is that all values were estimated using the positivity rate.

**Figure 6:**
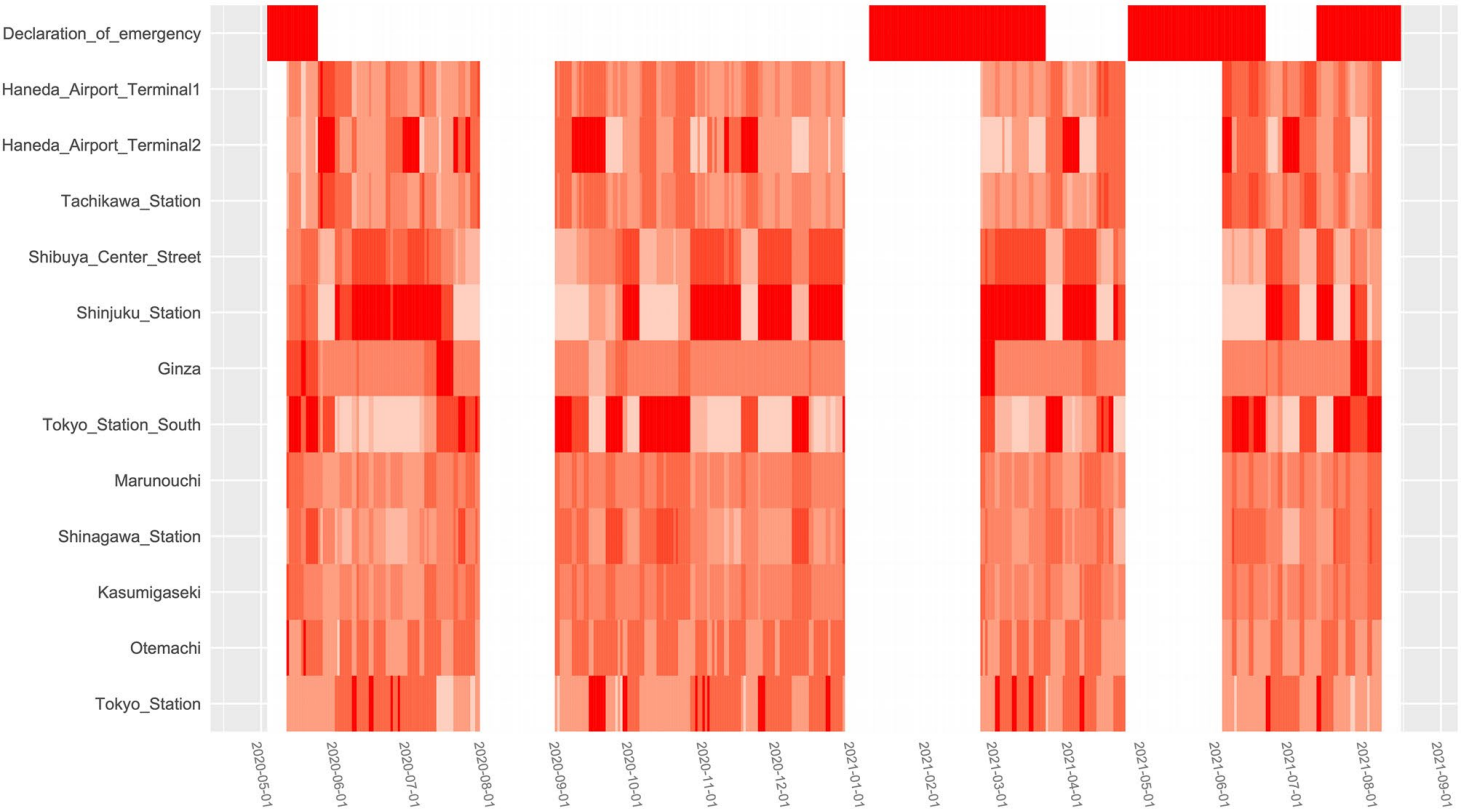
Semi-quantitative heatmap of the contribution of the number of people to effective reproduction number. The top row shows the period of the emergency declaration. Only the 2nd to 5th waves are shown. The darker the red colour, the greater the effect on the effective reproduction number (*R*_t_), and the lighter the red colour, the smaller the effect on *R*_t_. Periods that are not waves are shown in white. This figure was drawn in R version 4.0.3 (https://cran.ism.ac.jp/bin/windows/base/old/4.0.3/).

The *R*_t_ and 95% credible intervals estimated by model (2) are shown in Figs. 5A and B. The *R*_t_ values and estimates are in good agreement. The contribution of the number of people to the *R*_t_ during the wave periods is shown in Fig. 6. As the ratio of the increase/decrease in the number of people was only available after May, only the 2nd to 5th waves are shown. The top row shows the period of the emergency declaration. The contribution of the number of people to the *R*_t_ did not show common characteristics among the factor analysis groups. At HA Terminal 1, there was no largest contribution period, except for the period immediately after the first declaration of the SoE, and the contribution was relatively small under the declarations. On the contrary, at HA Terminal 2, there were periods when the contribution was the largest in each wave, and some of these periods coincided with consecutive holidays and the day before these holidays. All the periods with the largest contribution were not under the SoE, except for 3–7 June 2021. Although Tachikawa Station was not in the same group as HA Terminal 1, a similar trend was observed. This was because the category of the contribution of Tachikawa Station estimated from the number of cPCs and that of HA Terminal 1 estimated from the PR was the same, and vice versa. Although Shibuya Center Street did not have a period with the largest contribution, it had the longest period with the second-largest contribution. Shinjuku Station is the point with the longest period of the largest contribution to the *R*_t_. As in Shibuya, the contribution in the latter half of the second declaration was estimated to be quite large. Although the period of the largest contribution was small at Ginza, it had a moderate impact on the average. This is consistent with the fact that the number of people remains almost the same, except for the period immediately before and after the declaration. At Tokyo Station South, there was a mixture of periods with large and small contributions. The contribution was estimated to be the largest at the start of the second and third waves. Although Marunouchi and Kasumigaseki were different groups, similar trends were observed. The contribution at both was estimated to be relatively light to moderate. Although there was no period in which the contribution at Shinagawa Station was the largest, it seemed to be larger than that at Kasumigaseki, even in the same group. Otemachi had two periods with the largest contribution, although they were very short. It was suggested that the contribution at the other sites was moderate on average. At Tokyo Station, many periods with the largest contribution were observed, although the total period was short.

## Discussions

In this study, the *R*_t_ was estimated from two data points, the number of cPCs and PR. The EAP and waves were defined based on the *R*_t_. We estimated the contribution of the number of people to *R*_t_ at the 12 sites. Since COVID-19 is transmitted from human to human, the more contact opportunities there are, the more the number of infected people is expected to increase. These results qualitatively support this principle. However, it was also suggested that the contribution at each site was inconsistent, even if the number of people were similar.

This study included 366,894 individuals over the period 24 January 2020 to 13 September 2021. The period from onset to confirmed positive was available for 236,437 (64.4%) of individuals and 297 (0.1%) had a negative duration. The minimum and maximum values of the negative periods were -189 and -1, respectively, suggesting that there was a mixture of erroneous data entry and cases in which the positive result was confirmed by testing before the onset of the disease. Although the latter cases were not considered in this study, their influence on the results is not expected to be significant because of their small proportion. In addition, both the mean and standard deviation did not change significantly when the period of data used for the sensitivity analysis was changed. In this study, a 95% range was used. The reliability of the estimated distribution was not considered to be low, because there was no significant change in the mean and standard deviation, even when the 99% data range was used.

The most characteristic trend in the number of visitors was observed at HA Terminal 2, where peaks, except for 30 March and 27 June 2021, coincided with consecutive holidays, Obon holidays, or New Year holidays, during which there is a lot of travel and visiting parents’ homes. As for the non-holiday peaks, they occurred immediately after the declaration of the SoE was lifted, which may have caused a sharp increase in travel demand. In factor analysis, factor 1 was large in Kasumigaseki, Otemachi, Shinagawa Station, Tokyo Station, Ginza, Marunouchi, and Tokyo Station South, while factor 2 was large in Shibuya Center Street, Shinjuku Station, and Tachikawa Station. Factor 2 was about 0.2–0.3 in Ginza, Marunouchi, and Tokyo Station South. Since Kasumigaseki and Otemachi do not have downtown or nightlife spots, Factor 1 can be interpreted as a weekday, business, or office-associated factor. On the other hand, factor 2 can be considered as a holiday, office, shopping area, and nightlife spot associated factor, since Shibuya Center Street and Shinjuku Station have a mix of offices, shopping areas, and nightlife spots. The fact that factor 2 is not small in Ginza, Marunouchi, and Tokyo Station South can be interpreted consistently. The results of a sensitivity analysis using three factors did not change the interpretation of factors 1 and 2. Factor 3 was the largest at HA Terminal 2 and the next largest at HA Terminal 1 but had almost no loadings at the other locations. Therefore, Factor 3 was considered to be a factor representing holidays. While the loadings for factors 1 and 3 were relatively large at HA Terminal 1, the loadings for Factor 3 were extremely large at HA Terminal 2. This suggests that while HA Terminal 1 has a mix of both business use and holiday travel, HA Terminal 2 is mainly used for holiday travel. This is also supported by the fact that the peak of the trend in the number of people at HA Terminal 2 coincides with consecutive holidays and weekends.

In Japan, the number of inspections and cPCs decreased on Saturdays, Sundays, and holidays. Therefore, the *R*_t_ estimated from the number of cPCs was also cyclical, although it was somewhat mitigated by back-projection. In contrast, the *R*_t_ estimated from the PR was relatively unaffected by the number of inspections. However, the number of inspections is the sum of inspections conducted at the Health and Safety Research Center and medical institutions, and the PR at each institution was considered differently. Therefore, the influence of the number of infections on the PR is inevitable. To increase reliability, the period during which both *R*_t_ estimated by the two different data sets exceeded 1.0, was defined as the EAP. The number of people infected in the community was estimated by multiplying the PR of people with an unknown contact history (not considered to be close contacts of infected persons) by the number of susceptible people. Therefore, there is a certain degree of validity in estimating the *R*_t_ from the PR. In this study, we used the PR of the entire cPC instead of the PR of those with unknown contact history. However, there was no notable difference in the results, regardless of the selected rate. Waves were defined based on the EAP. As shown in Fig. 3C, the EAP is a series of short periods, and it is possible to distinguish the period when the infection is spreading from when it is not, by introducing the concept of waves. Since data on the number of people were available after 1 May, we were only able to observe the association between the number of people and the second to fifth waves. From the third wave onward, waves appear to occur as the number of people increased. For instance, when the trend of the number of people in Shinjuku station exceeded 250–300%, a wave started. Similarly, it seemed to have occurred when the trend in the number of people in Shibuya Centre Street exceeded 200–250%. The Tokyo Metropolitan Government requested that restaurants shorten their hours of operation from 30 July to 15 September 2020. Since no wave occurred during the period from 31 July to 30 August 2020, the shorter opening hours had a certain effect. However, it is not possible to evaluate its effectiveness in this study, since the number of people decreased during this period. The third and fourth waves ended with a declaration of an SoE. The number of people immediately decreased just before and after the declaration of the SoE, indicating that the declaration of the SoE had a significant impact. However, under the fourth declaration, we could not observe the same sharp drop in the number of people as in the second and third proclamations, nor did the wave come to an end. Under the fourth SoE, a combination of factors may have contributed to the failure to induce people to change their behaviour. First, individuals and society may have become accustomed to the declaration of an SoE as during the 255 days of 2021 within the study period, only 62 days are not under an SoE. Furthermore, there were only 33 days between the second and third emergency declarations, but the interval between the third and fourth declarations was even shorter, at 20 days. In addition to habituation, stress and other factors are also possible. Even though the Olympics Games were held under an SoE, people were not able to freely able to eat, drink in restaurants or travel. As a result, they had fewer ways to relieve stress, which may have prevented them from changing their behaviour. There may have been a variety of other factors, and as a result of a combination of those factors, the human flow may not have decreased as much as it did during the second and third SoE.

The number of people at the Kasumigaseki, Otemachi, and Shinagawa Stations, which were considered as one group by factor analysis, had a relatively light impact on the *R*_t_. This group was considered to have a strong weekday, business, or office-associated component. Therefore, it was suggested that offices are less likely to be a place of infection spread, although a few clusters have been reported. Only Tokyo Station has a period when the contribution is the largest, though they belong to the same group. Tokyo Station has a much higher number of train arrivals and departures. It is possible that some factors were not present at other stations. However, this study could not clarify this. Ginza, Marunouchi, and Tokyo Station South were grouped by factor analysis. They appeared to have periods with the largest contribution. Compared to Kasumigaseki, Otemachi, and Shinagawa Stations, they included downtown and nightlife spot associated factors, and this result was considered to be reasonable. There is also a concern about Tokyo Station, Ginza, Marunouchi, and Tokyo Station South. Since these areas are very close to each other, there is a possibility that the baseline population ratios might not be well estimated. Although the Open Data on Human Flows are based on a 1 km mesh of population, Tokyo Station, Marunouchi, and Tokyo Station South are all on the same mesh. Ginza is located in a neighbouring mesh. Therefore, these baseline population ratios may be unstable compared to other locations and may not match the actual situation. Shinjuku Station, Shibuya Center Street, and Tachikawa Station were classified as one group. Among these, the period with the largest contribution to the *R*_t_ was extremely long in Shinjuku Station. Of the 12 sites, Shinjuku Station had the longest period with the largest contribution. These results for Shinjuku station are considered to be reasonable since the factors associated with the downtown area and nightlife spots are extremely large in this group. It is interesting to note that the pattern of contribution to *R*_t_ for Shinjuku Station and Shibuya Centre Street is similar in the same group but completely different for Tachikawa Station. There may be some reasons for this, such as not attracting as many people from a wide area as the other two sites or having many residential areas. However, the reasons for this cannot be clarified by this study alone. HA Terminals 1 and 2 belong to independent groups. While the contribution of the number of people in Terminal 1 is relatively small, there are periods when the contribution is the largest in Terminal 2. Although Terminal 1 had factors associated with holidays, downtown, and nightlife spots, the loadings of factors associated with weekdays, business, and offices are much stronger. The situation was the opposite for Terminal 2. Therefore, this result was also considered natural. Almost all of the periods in which the contribution of HA Terminal 2 was the largest were not under the declaration of an SoE. It is thought that the infection spread due to a sudden increase in travellers after the declaration of the SoE was lifted. On 1 October 2020 Tokyo departures were added to the “Go To” travel campaign, which was a kind of state subvention for domestic travel. The holiday that followed was 3 November 2020, but it was not a consecutive holiday because 2 November was a weekday. Likely because of this, there was no significant increase in the trend of the number of people at HA Terminal 2. In addition, the contribution of the number of people at HA Terminal 2 was not large at this time. The next holiday was 23 November, which was a consecutive holiday together with Saturday and Sunday. During this consecutive holiday, the trend in the number of people at HA Terminal 2 exceeded 300% at the maximum. The contribution to the *R*_t_ was the largest among the 12 sites. Although it is impossible to obtain the number of people in the absence of the campaign, the possibility of the campaign spreading the infection cannot be denied. In fact, Anzai et al. examined the incidence rates of travel-associated and tourism-related infections and suggested that the “Go To” Travel campaign may have contributed to the increase in travel-related infections^11^. Although not a novel coronavirus, Petersen et al. found that air travel in the United States contributed to the rate of interregional transmission of influenza^12^, suggesting an association between travel and the spread of infection.

Interestingly, our results suggest that the magnitude of the contribution to the *R*_t_ may differ even when the number of people is similar. For example, the trend in the number of people at HA Terminal 2 was not significantly different between 23 November and 27 December 2020, both being at roughly 300%. However, the contribution to *R*_t_ was largest during the former period, while it was smaller during the latter period. The reason for this is unclear, but one reason may be that in the former period there were many trips for sightseeing, while in the latter period there were many trips for returning to parents’ homes. It is reasonable that there are fewer opportunities for human-to-human contact in trips for returning to parents’ homes than for sightseeing. Shinjuku Station is another example. The trend of the number of people in Shinjuku Station in the early stages of the 4th and 5th waves was generally the same, at roughly 250–300%. However, the contribution to the *R*_t_ at the beginning of the 4th wave was the largest, while the contribution at the beginning of the 5th wave was small. This difference can be attributed to the difference in the content of the second and third emergency declarations; in the second emergency declaration, the Tokyo Metropolitan Government requested restaurants and entertainment facilities with restaurant business licenses to shorten their business hours. In other words, the hours of operation were set from 5:00 to 20:00, and alcoholic beverages were served from 11:00 to 19:00. In contrast, the third emergency declaration requested restaurants and entertainment facilities that served alcoholic beverages or karaoke equipment to close their doors. After the third emergency declaration was lifted, priority measures to prevent the spread of new coronavirus infection were implemented, which allowed the serving of alcoholic beverages in restaurants and entertainment facilities with a restaurant business license, with some restrictions. The fourth emergency declaration was similar to the third one, and it was expected that the contribution at Shinjuku Station would decrease. However, it was the largest from 12 July to 18 and 26 July to 27, 2021. The reasons for this could not be clarified in this study. However, there may have been some behavioural changes in the vicinity, since July 21–25, 2021, is a consecutive holiday, and the Tokyo Olympics Games started on 21 July.

The model used in our study does not allow us to directly estimate the effect of the Olympics. Therefore, it is only a descriptive epidemiological discussion. Assuming that the period of the Olympics runs from the opening to closing ceremony, the Olympics ran from 23 July, 2021, to 8 August, 2021. To avoid an increase or decrease in the number of cPCs, only the *R*_t_ estimated based on PRs was focused on. The *R*_t_ continuously exceeded 1.0 from 7 to 16 July, and then exceeded 1.0 again on 21 July, and remained above 1.0 until 30 July. After that, it exceeded 1.0 on 8 and 9 August, and has been below 1.0 since then. One suggestion can also be made from human flow at the 12 sites. Neither the trend component alone nor the sum of the trend and residue showed any noticeable change in human flow during and before the Olympic period. On the contrary, there was a decrease in flow in the vicinity of 23 July and 8 August. Only HA Terminal 2 seen an increase in the flow of people during the same period. Perhaps, many of them watched the opening and closing ceremonies at home because they were not allowed to watch the Olympics in stadiums. Considering the information was obtained indirectly from the *R*_t_ and human flow, it is thought that the impact of the Olympics on the spread of infection was not significant. Of course, the results of this study do not provide any information on whether the end of the epidemic would have been accelerated if the Olympics had not been held. Therefore, since no direct results were obtained in this study, future study on the association between the Olympics and the infection situation is required.

The modes of transmission of SARS-CoV-2 are contact, droplet, and aerosol infections^13,14^. Since many restaurants and other establishments have alcohol disinfectant at the entrances and open the entrances to improve ventilation in Japan, special attention should be given to droplet infection. As many people wear masks when they go out, the risk of infection is highest when they remove their masks and engage in activities that generate droplets. One such activity is nightlife. Nagata et al. reported that changes in mobility were more strongly associated with epidemics in nightlife spots than in workplaces and residential areas^15^. Nakajo et al. proposed a new method for estimating the *R*_t_ in real-time and reported an association between the epidemic and social measures in Osaka. During the second wave restrictions were requested in Osaka Prefecture, such as self-regulation of social events involving the consumption of alcohol by more than four people and voluntary closures of restaurants and bars in certain areas. However, these alone may not be sufficient to reduce the *R*_t_ to less than 1.0^16^. Nakanishi et al. suggested an association between the night-time population in Tokyo and infection spread^17^. Since we used the number of people at 15:00 in this study, we cannot relate findings to nightlife. However, the results of previous studies are consistent with the interpretation of our factor analysis results. It was indicated that the rate of travel to restaurants, cafes, shopping centres, theme parks, etc. was well associated with the *R*_t_ during a high number of infected people under a declared SoE, and that the population staying overnight was more accurately related to temporal changes in the effective reproduction number *R*_t_ when the epidemic size was small^18,19^. In this study, we only used the population at a location and did not consider the destination, which is a future consideration. In Tokyo, as of 21 September, 2021 more than 86% of the population aged 65 and above received two doses of vaccine, and about 55% of the total population had received two doses of vaccine^20^. It was indicated that the vaccine reduced the *R*_t_ by 20% at the end of August compared to 21 June 2021^18,19^. Thus, the fifth wave may have ended because of a combination of reduced human flow and vaccine efficacy.

This study has several limitations. Firstly, it does not consider spatial distribution of infected people. When the number of infections is low, it is expected that many infected people in so-called clusters have a known contact history. The model assumes a uniform population distribution of infected people. Therefore, the results may not be valid, especially for a small number of infected individuals. However, a considerable number of cPCs were reported after the third wave, and for this study, it does not seem to be enough to deny the estimated *R*_t_. Another limitation is that age was not considered. It has been suggested that susceptibility varies with age^21^. Since age-related characteristics such as vaccination coverage and behavioural patterns may influence infection, it would be better to consider the age structure. In the fifth wave, the proportion of confirmed positive individuals aged 65 years and older decreased over time. However, since we could not find any age-related data on human flow, we consider this a future research area. The next limitation is that the *R*_t_ of Tokyo is investigated using the number of people at 12 sites. There are many other stations in Tokyo, such as Ikebukuro Station, which is second only to the Shinjuku Station and Shibuya Stations. However, we could not obtain data on the number of users. Although the number of people outside the 12 points is represented by a single coefficient, the *R*_t_ estimated by model (2) is a good fit. The final limitation was that the theoretical basis for adopting model (2) is not very strong. There were papers in which the dependency of population size was assumed to be exponential (e.g. Baker et al.^22^) though the model itself was quite different from ours. However, we consider our research as one attempt. The improvement of the model is also one of the issues to be considered in future research.

In this study, we estimated the *R*_t_ at 12 locations and semi-quantified the contribution of the number of people to the *R*_t_ in Tokyo. It is the first study to report on the association between human flow and the *R*_t_ in Tokyo over a long-term period. In some cases, the magnitude of the contribution to the *R*_t_ by human flow was inconsistent, even when human flow was similar. This study could not clarify the reason for this. Further research to allow more effective control of human flow is recommended.

## Materials and methods

We used the data published by NTT DOCOMO. The data provide the estimated population based on mobile device location^23^. Daily population change as of 15:00 is freely available on the website^24^. These data show the population change from the previous day at several points in each prefecture. Data for four or more sites are shown for Tokyo, Kanagawa, Ibaraki, Osaka, Fukuoka, and Hokkaido. We examined data for Tokyo, where sufficient information on the number of cPCs per day, the number of tests, and the age groups of the cPCs were available^25^. The 95% data range was used because some outliers were identified in the period between the onset and confirmation of a positive test result. The distribution with the highest likelihood when fitting the gamma, lognormal, and Weibull distributions was used. For the incubation period, we used a lognormal distribution with a mean of 5.8 and a standard deviation of 3.1, as in previous studies^26^. $C(t)$ is defined as the number of cPCs at time $t$. $C(t)$ includes a delay in the period from the onset to cPCs and that of incubation period. To clarify the dynamics of epidemic, the number of infected individuals at time $t$, $\widehat{C}(t)$ was estimated by back-projecting both distributions into $C(t)$. The *R*_t_ was estimated by applying the following reproduction Eq. (1) to $\widehat{C}(t)$:

1 $$\widehat{E}\left(\widehat{C}(t)\right)={R}_{t}{\sum }_{\tau =1}^{t-1}g(\tau )\widehat{C}\left(t-\tau \right),$$

where $g(\tau )$ is the serial interval, and we used the Weibull distribution with median, mean, and standard deviation of 4.6, 4.8, and 2.9 days, respectively, as reported by Nishiura et al.^27^. It was also assumed that $\widehat{C}(t)$ follows a Poisson distribution with $\widehat{E}\left(\widehat{C}(t)\right)$ as the mean (the posterior distribution was assumed to be a gamma distribution with a scale parameter of 1.0). Because the number of inspections fluctuated greatly over time, we estimated the *R*_t_ in two ways, using not only the number of cPCs but also the positivity rate per day for $C(t)$. In this study, the EAP was defined as the period when the *R*_t_ estimated by two different data exceeded 1.0. Furthermore, the periods of the first, second, third, fourth, and fifth waves were defined from the EAP.

The number of people per day at each site, in which on 30 April 2020 was defined as 100%, was calculated by using data on the ratio of increase/decrease to the previous day^24^. In Tokyo, an increase or decrease in the number of people at 12 locations (Tokyo station, Otemachi, Marunouchi, Tokyo station south, Kasumigaseki, Ginza, Shinjuku Station, Shibuya Center Street, Shinagawa Station, HA Terminal 1, and HA Terminal 2) were available. The ratio of the number of people in each area on 30 April was estimated from the national human flow open data published by the Ministry of Land, Infrastructure, Transport and Tourism^28^. The rate of increase in the decrease in the number of people was based on the number of people at each site at 15:00. In addition, the monthly converted population for each 1 km mesh in Japan is entered into the Open Data on Human Flows. Therefore, we first combined the tertiary mesh data of the national standard regional mesh (approximately 1 km square)^29^ with the National Land Information Railway data^30^ and then overlaid the open data on human flows to obtain the coordinates of 12 points. Next, we extracted noon (11:00 to 14:00 average) weekday and holiday populations at the relevant coordinates for the 12 sites from May to December 2020. Similarly, the rate of increase/decrease was calculated monthly from May to December for each weekday and holiday. Finally, a multiple regression analysis using the rate of increase/decrease and the classification of weekdays of holidays as variables was used to estimate the population ratio on 30 April 2020 for each location. QGIS version 3.16 was used to superimpose the geographic information. In Tokyo, 12 locations were examined for the rate of increase or decrease, but since some locations showed similar trends, factor analysis with Quartimin rotation was used to examine locations with similar trends. In addition, the association between the *R*_t_ and the number of people was examined using Eq. (2):

2 $${R}_{l,t}={C}_{l}{e}^{{\epsilon }_{l}}\prod_{i}{({B}_{i}{M}_{i,t})}^{{k}_{j,l,T}}$$

where $C$ is a normalisation constant, and $i$ and $j$ denote the number of locations (from 1 to 12) and the number of groups classified by factor analysis (from 1 to 5), respectively. $l$ is the type of data used for estimating the *R*_t_ (i.e. the number of cPCs or PRs). ${B}_{i}$ is the population ratio at point $i$ as of 30 April as estimated above. ${M}_{i,t}$ is the increase or decrease in ratio of the number of people at point $i$ at time $t$. ${k}_{j,l,T}$ is assumed to be dependent on the number of people at the group $j$ in week $T$ since ${k}_{j,l,T}$ is also assumed to remain unchanged over the week. As a sensitivity analysis, the start date was varied from 30 April to 6 May and the date with the smallest WAIC was selected. It was also assumed that ${\varepsilon }_{l}$ follows a normal distribution with mean 0 and an unknown parameter ${\sigma }_{l}$ as the standard deviation. The values of ${({B}_{i}{M}_{i,t})}^{{k}_{j,l,T}}$ based on the number of cPCs were arranged in descending order to create an ordinal variable. Then, we assigned a score of 20, 15, 10, or 5 to each of the first to third, fourth to sixth, seventh to ninth, and tenth to twelfth places, respectively. Finally, we semi-quantified the contribution of human flow at each location to the *R*_t_ by adding up the scores for each location. In this study, the parameters were estimated using the Markov chain Monte Carlo method. The number of iterations, chains, and burn-in was 20,000, 4, and 10,000, respectively. The Rhat statistic of Gelman-rubin was determined to converge because it is less than 1.1. The increase and decrease in the number of people had a cyclic nature over one week. Because the figure was very difficult to read, we used the method by Cleveland et al.^31^ to remove the periodic component and observed only the trend of the number of people. However, the raw data are used in Eq. (2). R version 4.0.3 was used for all statistical analyses in this study. All methods were carried out in accordance with the relevant guidelines and regulations governing the use of public data sources.

## Data Availability

Ratio and trend of increase/decrease in the number of people at 12 sites are available from "https://mobaku.jp/covid-19/". All data on COVID-19 in Tokyo are available from "https://stopcovid19.metro.tokyo.lg.jp/". The tertiary mesh of the national standard regional mesh (about 1 km square), railroad data, and the national open data on human flows are data made public by the Ministry of Land, Infrastructure, Transport and Tourism. They were available after registration at the website (https://www.geospatial.jp/gp_front/). All the data used in this study are publicly available data.

## Abbreviations

SoE: The state of emergency
*R*_t_: The effective reproduction number
cPCs: Confirmed positive cases
PRs: Positivity rates
HA: Haneda Airport
EAP: Epidemic acceleration period (defined in Materials and Methods section)
